# Haptoglobin is dispensable for haemoglobin uptake by *Trypanosoma brucei*

**DOI:** 10.3389/fimmu.2024.1441131

**Published:** 2024-07-18

**Authors:** Eva Horáková, Marek Vrbacký, Martina Tesařová, Eva Stříbrná, Jan Pilný, Zuzana Vavrušková, Marie Vancová, Roman Sobotka, Julius Lukeš, Jan Perner

**Affiliations:** ^1^ Institute of Parasitology, Biology Centre, Czech Academy of Sciences, České Budějovice, Czechia; ^2^ Centre Algatech, Institute of Microbiology, Czech Academy of Sciences, Třeboň, Czechia; ^3^ Institute of Physiology, Czech Academy of Sciences, Prague, Czechia; ^4^ Faculty of Science, University of South Bohemia, České Budějovice, Czechia

**Keywords:** haptoglobin (Hp), infection, *Trypanosoma*, haemoglobin (Hb), blood markers, acute phase protein

## Abstract

Haptoglobin is a plasma protein of mammals that plays a crucial role in vascular homeostasis by binding free haemoglobin released from ruptured red blood cells. *Trypanosoma brucei* can exploit this by internalising haptoglobin-haemoglobin complex to acquire host haem. Here, we investigated the impact of haptoglobin deficiency (Hp-/-) on *T. brucei brucei* infection and the parasite´s capacity to internalise haemoglobin in a Hp-/- mouse model. The infected Hp-/- mice exhibited normal disease progression, with minimal weight loss and no apparent organ pathology, similarly to control mice. While the proteomic profile of mouse sera significantly changed in response to *T. b. brucei*, no differences in the infection response markers of blood plasma between Hp-/- and control Black mice were observed. Similarly, very few quantitative differences were observed between the proteomes of parasites harvested from Hp-/- and Black mice, including both endogenous proteins and internalised host proteins. While haptoglobin was indeed absent from parasites isolated from Hp-/-mice, haemoglobin peptides were unexpectedly detected in parasites from both Hp-/- and Black mice. Combined, the data support the dispensability of haptoglobin for haemoglobin internalisation by *T. b. brucei* during infection in mice. Since the trypanosomes knock-outs for their haptoglobin-haemoglobin receptor (HpHbR) internalised significantly less haemoglobin from Hp-/- mice compared to those isolated from Black mice, it suggests that *T. b. brucei* employs also an HpHbR-independent haptoglobin-mediated mode for haemoglobin internalisation. Our study reveals a so-far hidden flexibility of haemoglobin acquisition by *T. b. brucei* and offers novel insights into alternative haemoglobin uptake pathways.

## 1 Introduction

**Figure 1:**
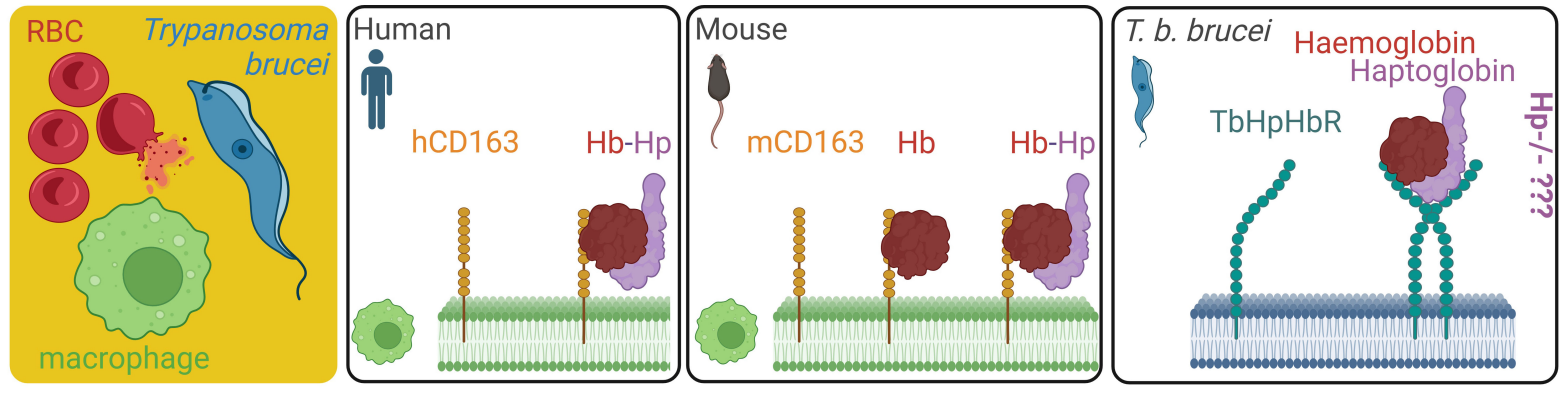
Overview of haemoglobin scavenging systems in human macrophages, mouse macrophages, and *Trypanosoma b. brucei* upon haemolysis. The source of extracellular haemoglobin in blood plasma is mostly ruptured red blood cells, setting a competition between the endogenous haemoglobin clearance mechanisms mediated by mice macrophages, and the haemoglobin salvage pathway of *T. b. brucei* in the infected mice. While we know that the mouse macrophages can bind free haemoglobin by the CD163 homologue, the impact of haptoglobin deficiency in host mice (Hp-/-) on *T. b. brucei* capability to internalise haemoglobin and retain infectivity is unknown. RBC, red blood cell; TbHpHbR, *T. brucei* haptoglobin-haemoglobin receptor; mCD163, mouse CD163 receptor; hCD163, human CD163 receptor.

African trypanosomes belonging to the complex of *Trypanosoma brucei* ecotypes are responsible for a number of serious diseases in humans and cattle (1). Being haem auxotrophs, trypanosomes cannot build this essential molecule from simpler building blocks via an endogenous haem biosynthetic pathway (2, 3). They need to secure the haem cofactors from external sources, i.e. from haemoglobin of their vertebrate hosts (4). *Trypanosoma b. brucei* is an extracellular parasite that predominantly localises in mammalian blood, where it multiplies as a bloodstream stage. To acquire haem, the bloodstream trypanosomes capture and internalise haemoglobin released from the lysed red blood cells, with haemolysis being an event concomitant to the *T. b. brucei* infection (5). The parasites internalise haemoglobin via recognition of the haptoglobin-haemoglobin (HpHb) complex, which is spontaneously formed in blood plasma. The interaction of the HpHb complex with *T. b. brucei* is mediated by a dedicated haptoglobin-haemoglobin receptor (HpHbR) expressed on the cell membrane of the bloodstream stage (6) and conserved across the *Trypanosoma* lineage (7–9). Concurrently, the HpHb complexes are sequestered from the blood by CD163-mediated clearance, i.e. the host self-defence mechanisms against an excessive build-up of extracellular haemoglobin (10). Deployment of HpHbR and CD163 by the parasite and the host, respectively, creates a micro-environment highly competitive for extracellular haemoglobin capture. This tug-of-war between the uptake of haemoglobin by trypanosomes and the host’s clearance mechanism represents a remarkable act of inter-organismal molecular interplay (**Figure 1**).

Using an Hp-deficient mouse model, we elicited a new experimental framework, where the parasite-host interaction takes place in the background with no Hp-mediated processes. While the human homologue of CD163 binds only the HpHb complex and does not interact with free uncomplexed haemoglobin (10), the mouse homologue of CD163 binds both free haemoglobin as well as the HpHb complex (11). Since we wondered how the absence of plasma Hp affects haem biology and pathogenesis of *T. b. brucei* in mice, we aimed to address the following questions: i) what are the collateral effects of Hp deficiency on the blood plasma proteome (i.e. the proteomic landscape where the parasite-host interaction occurs)? ii) can *T. b. brucei* survive and proliferate in the blood of an Hp-deficient mouse? iii) can the parasite internalise from the mouse bloodstream and make efficient use of haemoglobin uncomplexed with Hp?

To address these questions, we have infected haptoglobin knock-out (Hp-/-) mice with *T. b. brucei* and performed a comprehensive analysis of this parasite-host system. We have characterised the blood plasma proteome of Hp-/- mice, followed the dynamics of their trypanosome infection, and examined the parasite’s strategies for haemoglobin acquisition in an Hp-lacking host. By showing that *T. b. brucei* not only survives but also replicates in the bloodstream of Hp-/- mice, our data challenge the conventional paradigm, in which Hp plays an essential role during infection. The nuanced interplay between this parasitic flagellate and its host, the latter lacking a molecule considered critical for the parasite’s survival, yet again demonstrates the enormous plasticity of *T. b. brucei*, which has evolved (an) alternative pathway(s) for haemoglobin uptake, revealing a built-in versatility in its survival strategies. This research not only improves our understanding of the molecular intricacies of the *T. b. brucei* infection, but also challenges established dogmas about the key role of Hp in host-parasite interactions.

## 2 Results

### 2.1 Haptoglobin knock-out mice display complex quantitative proteomic differences in blood sera

**Figure 2:**
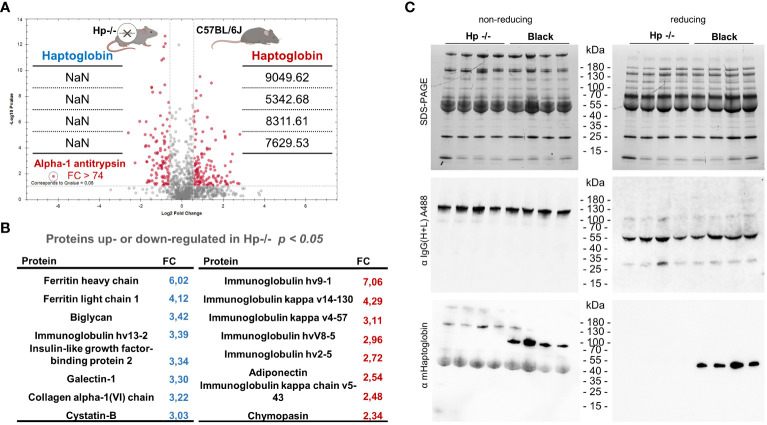
Proteomics profiling of blood plasma from Hp-/- and Black mice. **(A)** A volcano plot of mice proteins identified in the plasma of Hp-/- and control Black mice (C57BL/6J), FDR = 0.01. Note that the level of haptoglobin (MS2 intensities) is detected only in Black mice, confirming the absence of Hp in the plasma of Hp-/- mice. Data are derived from four independent replicates; NaN = not identified. **(B)** A list of proteins that were either more or less abundant in the blood plasma from Hp-/- mice compared to Black mice; n = 4, t-test *p < 0.05*. *A* full list of identified proteins is available as **Supplementary Data S1**. **(C)** SDS-PAGE separation of mouse blood plasma proteins and western blot analysis using primary antibodies, fluorescently labelled with Alexa 488 (A488), against the heavy and light chains of mouse IgG, showing little, if any, impact of Hp deficiency on the amount of circulating IgGs. Please see individual heavy and light chains under reducing conditions. The bottom western blot using anti-Hp antibodies unequivocally confirms full Hp deficiency in Hp-/- mice.

Mice deficient for haptoglobin (Hp-\-) were generated by directed excision of the *haptoglobin* gene (Acc. no: Uniprot Q61646) by homologous recombination in the C57BL/6JRj genetic background (Lim et al., 1998). Following the cross of heterozygous parents, we screened the offspring for homozygosity and verified the target gene deletion (**Supplementary Figure S1**). Using label-free proteomics of plasma acquired from Hp-/- and control Black mice prior to infection, we confirmed a complete lack of the Hp protein in the former mice (**Figure 2A**), which was concomitant with an additional quantitative disbalance in the blood plasma proteome (**Figure 2B**). For example, α-1 anti-trypsin was found markedly (Fold Change > 74) elevated in the sera of Hp-/- mice, together with both heavy and light ferritin chains (**Figures 2A, B**). A full list of identified proteins is available as **Supplementary Data S1**. *Vice versa*, dozens of proteins were less abundant in the sera of Hp-/- mice, with certain immunoglobulin chains being particularly strongly down-regulated (**Figure 2B**).

To elaborate on these observations, we used western blot analysis to validate haptoglobin deficiency in Hp-/- mice and to determine the level of circulatory immunoglobulin IgGs in the sera of Hp-/- mice. Using fluorescently labelled anti-mouse IgGs, reacting with the heavy and light chains of mouse IgG, we revealed no net change in circulating IgGs in Hp-/- mice compared to Black mice (**Figure 2C**). Note that the level of haemoglobin chains also remained unaffected in the plasma of Hp-/- mice, indicating no increased haemolysis or elevated free haemoglobin levels. Collectively, these proteomics data validate the full Hp deficiency in Hp-/- mice and provide a perspective of the proteomic landscape of the blood plasma in the mouse models used in this study.

### 2.2 *Trypanosoma b. brucei* undergoes a typical parasitic wave in Hp-/- mice

**Figure 3:**
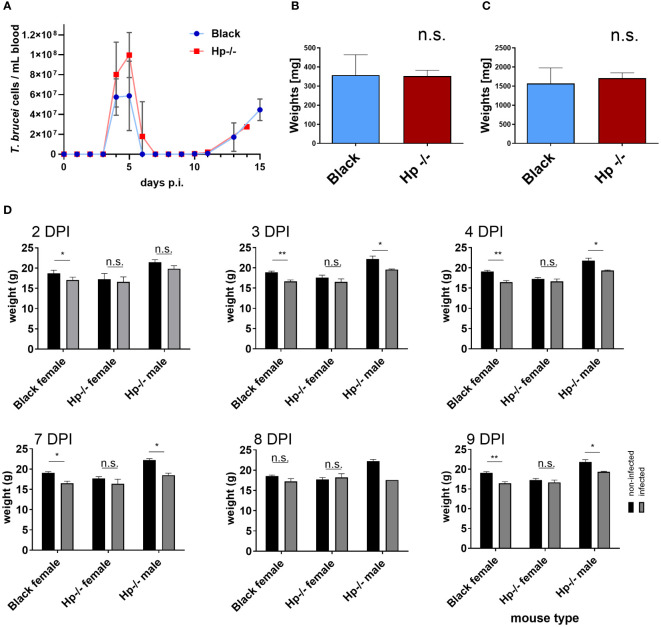
*Trypanosoma b. brucei* infection in Black and Hp-/- mice. **(A)** The bloodstream trypanosomes were injected intraperitoneally into Black (blue line) and Hp-/- mice (red line) and the resulting parasitaemia was monitored daily; n = 8, mean and SD are shown. **(B, C)** Bar graphs of weights of spleen **(B)** and liver **(C)** from Black and Hp-/- mice obtained at day 4 post-infection (DPI); n = 4, mean and standard errors of the means (SEM) are shown. Representative images of the organs are shown as **Supplementary Figure S2**. **(D)** Weights of non-infected (black bars) and infected mice (grey bars) are shown at the indicated DPI; n ≥ 4, means and SEMs are shown; t test: * = p < 0.05, ** = p < 0.01.

To evaluate the impact of a dysregulated blood plasma proteome on the course of *T. b. brucei* infection, we performed a comparative infection experiment in Hp-/- and Black mice and monitored the progression of parasitaemia. Trypanosomes were found not only to be viable in the Hp-/- mice bloodstream, but also progressed towards the parasitic wave, which peaked on day 5 post-infection, followed by swift clearance of the parasites from the bloodstream, similarly to the control mice (**Figure 3A**). In both mice strains, we documented no spleen or liver pathologies, as indicated by organ weight, during the parasitaemia peak (**Figure 3B**; **Supplementary Figure S2**). While the Black control mice (C57BL/6J) responded to trypanosomes with a partial weight loss, their Hp-/- counterparts displayed only a minor and insignificant weight loss in females, and a partial weight loss in males (**Figure 3C**). These data reveal that Hp-/- mice not only remain susceptible to *T. b. brucei*, but also retain mechanisms curbing excessive parasite load, despite inherent changes to the blood plasma proteome, including possible subtle immunoglobulin dysregulation.

### 2.3 Bloodstream stage acquires haemoglobin in Hp-/- mice

**Figure 4:**
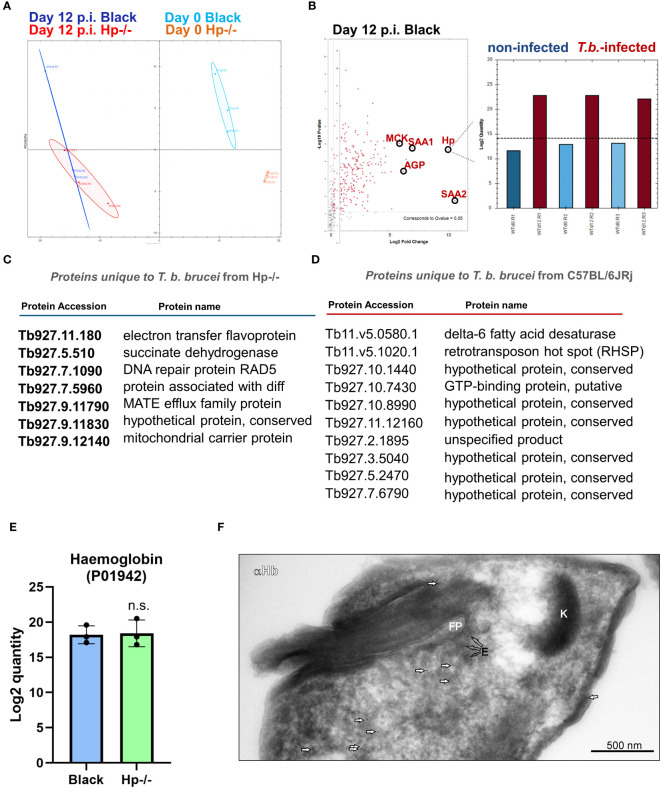
Dual proteomic analysis of plasma from non-infected and infected mice, and from *T. b. brucei* isolated from mice blood. **(A)** Principal component analysis of proteomics data of plasma obtained from Hp-/- and Black mice before and after infection. **(B)** A volcano plot of proteomics data of sera from control mice 12 days post-infection (p.i.), with significantly (Q value = 0.05) increased quantity of proteins responsive to the *T. b. brucei* infection. Haptoglobin (Hp) was identified in the plasma from Black mice as a *T. b. brucei* response marker with its level consistently elevated during infection (right inset) (n = 3). SAA1 and SSA2, serum amyloid A-1 and A-2 proteins, respectively; AGP, α(1)-acid glycoprotein; MCK, creatine kinase M type. **(C)** A list of proteins identified in *T. b. brucei* isolated from Hp-/- mice (n = 3), and not identified in parasites isolated from Black mice (n = 3). **(D)** A list of proteins identified in *T. b. brucei* isolated from Black mice (n = 3) and not identified in parasites isolated from Hp-/- mice (n = 3). **(E)** A proteomic quantification of mouse α-subunit haemoglobin (Acc. No. P01942) in *T. b. brucei* isolated from infected Black or Hp-/- mice. **(F)** Wild type parasites were purified from the blood of Hp-/- mice and imaged by transmission electron microscopy. Mouse haemoglobin was detected using α-haemoglobin antibodies and visualised with 10-nm gold nanoparticles coupled to protein A (arrows). Kinetoplast (K), flagellar pocket (FP), endosomes **(E)**. Negative control, represented by an application of gold-labelled protein A only, produced no signal (data not shown), confirming the specificity of haemoglobin detection.

To determine the responsive proteomic signatures of the Hp-/- and control mice sera to infection, as well as the behaviour of the *T. b. brucei* bloodstream stage in this specific host environment (Hp-/-), we concurrently harvested parasites and the mice blood plasma during the second parasitaemia wave, i.e. 12 days post-infection. The proteomic analysis of mice plasma samples revealed profound changes as a response to *T. b. brucei* infection in both Hp-/- and control mice (**Figure 4A**). In the latter, the parasites stimulated the expression of Hp, making it one of the most responsive plasma proteins to infection (**Figure 4B**). This enhances the validity of our model system, as it broadens the quantitative differences between Hp-/- mice lacking any Hp, and control mice with induced Hp in their sera. Furthermore, serum amyloid A-1 and A-2 proteins (SAA1 and SAA2), creatine kinase M type (MCK), and α(1)-acid glycoprotein (AGP) were plasma markers significantly upregulated in response to infection in Black mice (**Figure 4B**). In Black mice, *T. b. brucei* infection also induced expression of a large battery of plasma proteins, which were present exclusively in the plasma of infected mice, encompassing proteases (Napsin, Legumain, Cathepsin G, Cathepsin O), enzymes of the tyrosine degradation pathway (homogentisate 1,2-dioxygenase; 4-hydroxyphenylpyruvate dioxygenase), and a chitinase to name a few (**Supplementary Data S2**). In Hp-/- mice, in contrast, *T. b. brucei* infection caused very little plasma protein upregulation (**Supplementary Data S2**).

The comparison of proteomic spectra of the bloodstream stage trypanosomes, isolated concurrently from the Hp-/- and Black mice, revealed very few statistically significant differences (FC ≥ 5) (**Supplementary Figure S3**, **Supplementary Data S3**). Still, 7 and 10 *T. b. brucei* proteins were only present in parasites isolated from Hp-/- or Black mice, respectively (**Figures 4C, D**; **Supplementary Data S3**).When host proteins internalised by trypanosomes infecting Hp-/- mice were inspected, apart from the prominent absence of Hp, very few other proteins stood out, as if in these mice the parasites experienced no nutritional deprivation. Specifically, we observed no difference in the quantity of host haemoglobin in parasites isolated from Black and Hp-/- mice (**Figure 4E**), irrespective of whether host mice contained or lacked Hp in their blood. To investigate the origin of the haemoglobin peptides, we measured the MS2 intensities. While peptides derived from the mouse haemoglobin beta chain were exclusively of mouse origin, peptides from the alpha chain were a mixture of mouse and bovine haemoglobin. However, the vast majority originated from the mouse (MS2 intensities: mouse Hb 3.2 × 10^6 vs. bovine Hb 1.5 × 10^5). These data suggest that *T. b. brucei* can carry over a limited amount of host macromolecules from cultures. However, most haemoglobin molecules found in *T. b. brucei* 12 days post-infection are of immediate host origin. This validates our interpretation of similar haemoglobin uptake by *T. b. bruc*ei parasites in Black and Hp-/- mice. The full list of proteins, of both host and parasite origins, identified by proteomics in wild type *T. b. brucei* parasitising Hp-/- and Black mice is described in **Supplementary Data S3**. Note that the level of *T. b. brucei* HpHbR is identical in parasites isolated from Hp-/- and Black mice (**Supplementary Data S3**). To support our observation of identical quantities of haemoglobin in parasites isolated from Black and Hp-/- mice, immunodetection by western blotting (**Supplementary Figure S4**) and on parasite cryo-sections was carried out (**Figure 4F**). The latter confirms the internalisation of host haemoglobin at high resolution, the signal being localised to the cytoplasmic space near the flagellar pocket, i.e. a region characteristic for its endocytic/exocytic activity (**Figure 4F**). Altogether, these data reveal that *T. b. brucei* can induce a profound plasma proteome change in mice and that the parasite is endowed with the capacity to internalise haemoglobin without the prerequisite to form complexes with Hp in the blood.

### 2.4 *T. b. brucei* lacks haem *o* during infection of Hp-/- mice

**Figure 5:**
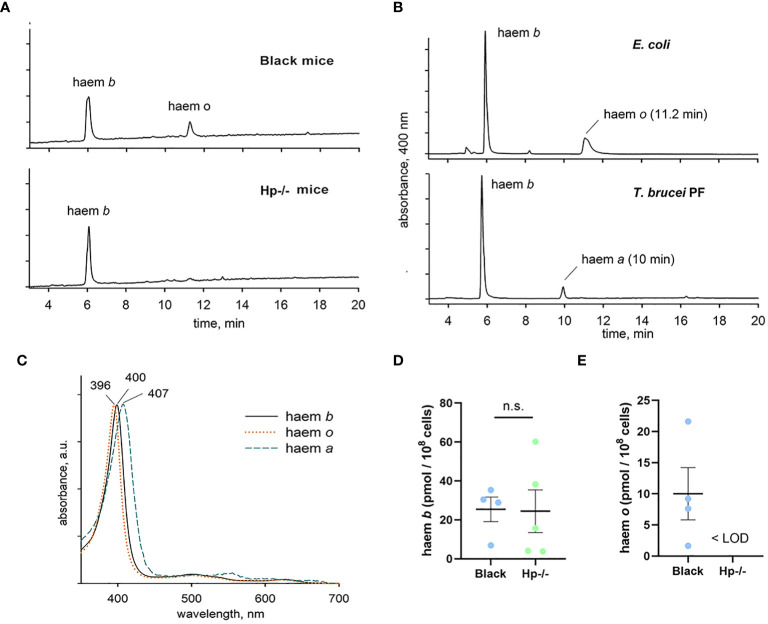
Identification and quantification of haem species in *T. b. brucei*. **(A)** Parasites isolated from Black mice contain both haem *b* and *o*, while haem *o* is virtually absent in cells isolated from Hp -/- mice. **(B)** Haem species in the bloodstream stage of *T. b. brucei* were identified using control extracts from *Escherichia coli* and from the procyclic stage of *T. b. brucei*. The retention time and absorbance spectra **(C)** were used to determine the authenticity of the haem species, showing identical levels of haem *b* **(D)** between the bloodstream of *T. b. brucei* isolated from Hp-/- and Black mice, and the presence of haem *o* **(E)** only in the bloodstream of *T. b. brucei* isolated from Black mice; LOD, limit of detection.

In order to confirm that *T. b. brucei* can accumulate haem cofactors in both Black and Hp-/- mice, acetone extracts of purified parasites were analysed by HPLC. Intriguingly, we detected two haem peaks in *T. b. brucei* isolated from Black mice, but only a single (polar) haem in cells from Hp-/- mice (**Figure 5A**). As controls, extracts from the procyclic stage of *T. b. brucei* known to contain haem *b* and *a* (12)and from *Escherichia coli* known to contain haem *b* and *o* (13) were used to assign haem forms in the bloodstream stage of *T. b. brucei*. Both retention time and spectra (**Figures 5B, C**) unambiguously showed that the parasites from Black mice contained both haem *b* and *o.* Although the content of haem *b* varied, this measurement further supports the view that there was no deviation in haem *b* availability between the parasites isolated from Black and Hp-/- mice (**Figure 5D**). On the other hand, (farnesylated) haem *o* was present in parasites isolated from Black mice but was below the limit of detection in those purified from Hp-/- mice (**Figure 5E**).

### 2.5 *T. b. brucei* internalises only a fraction of host haemoglobin via HpHbR *in vivo*

**Figure 6:**
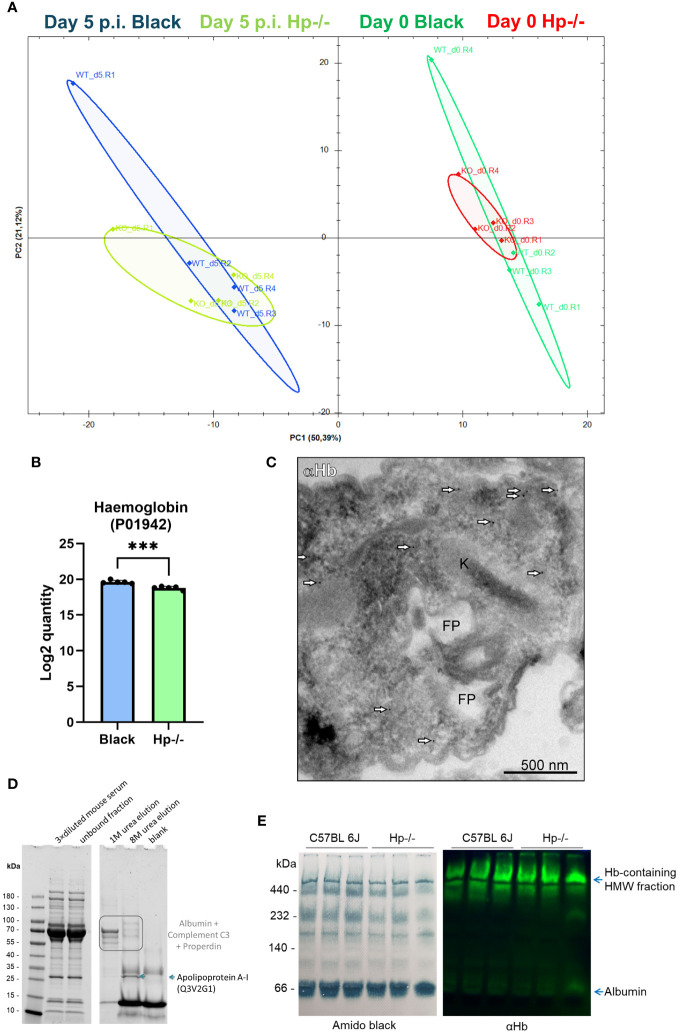
Assessment of haemoglobin internalisation by HpHbR-KO *T. (b) brucei* isolated from Hp-/- and Black mice. **(A)** Principal component analysis of proteomics data of plasma obtained from the Hp-/- and Black mice before and after infection (5 days post infection) by the HpHbR-KO parasites, n = 4. **(B)** The bar graph shows the Log2 quantity of haemoglobin internalised by HpHbR parasites isolated from Black and Hp-/- mice, as determined by proteomics. Means and SDs are shown; n = 5; t test *p* = 0.0004. **(C)** The HpHbR-KO parasites were isolated from the blood of Hp-/- mice for imaging by transmission electron microscopy coupled with immunodetection. Mouse haemoglobin was detected using α-haemoglobin antibodies and visualised through 10 nm gold nanoparticles-coupled with protein A (arrowheads). Kinetoplast (K), flagellar pocket (FP). **(D)** Reducing SDS-PAGE of haemoglobin pull-downs from mouse sera visualised in a stain-free mode; left gel indicates protein separation of starting material (diluted mice sera) and the supernatant after incubation with haemoglobin beads; right gel shows elution fraction from the beads: first two lanes show elution from haemoglobin beads after incubation with mice sera; blank indicates 8M elution from haemoglobin-beads after incubation with PBS only, instead of mice sera. **(E)** Membranes of electrophoretically separated serum proteins under native conditions; left, amido black staining for proteins; right, immunoblot against mouse haemoglobin; HMW, high molecular weight.

Having established that the bloodstream stage acquires haem(oglobin) even from Hp-/- mice, we sought to reveal mechanisms behind this process. Therefore, we generated a haptoglobin-haemoglobin receptor (HpHbR) knock out cell line (HpHbR-KO) and subjected these parasites to the workflow described above. The HpHbR-KO *T. b. brucei* induced proteomic signatures in the blood plasma of Black mice 5 days post-infection, during the first parasitaemia peak (14), with clear segregation of mouse plasma proteomes prior to and post-infection (**Figure 6A**). Again, Hp was determined as an infection response marker of mouse plasma, even for the HpHbR-KO parasites (**Supplementary Figure S5**). The full list of plasma response markers to the HpHbR-KO infection in both Hp-/- and Black mice is described in **Supplementary Data S4**.

Furthermore, the proteomics of HpHbR-KO revealed that parasites isolated from Black and Hp-/- mice contained virtually identical (FC ≥ 5) spectra of endogenous and host proteins (**Supplementary Figure S6**). However, inspection of specific peptides of mouse haemoglobin revealed a statistically significant decrease in the amount of internalised haemoglobin in the HpHbR-KO parasites isolated from Hp-/- mice (**Figure 6B**). These data show that trypanosomes lacking HpHbR internalise lower amounts of mouse haemoglobin when in Hp-/- mice, but still contain a majority of total haemoglobin found in the parasites isolated from Black mice. This indicates that Hp also promotes internalisation of host haemoglobin via an HpHbR-independent route and that most of it is acquired without the need to exploit Hp-haemoglobin binding capacity. The full list of host and parasite proteins identified in wild type *T. b. brucei* isolated from Hp-/- and Black mice is available in **Supplementary Data S5**. To further confirm genuine internalisation of haemoglobin, and to rule out possible non-specific surface adherence, we subjected the HpHbR-KO parasites isolated from Hp-/- mice to immunogold labelling. Similarly to wild type trypanosomes, the HpHbR-KOs localised host haemoglobin in the vicinity of the flagellar pocket in these HpHbR parasites when in Hp-/- mice (**Figure 6C**).

To elaborate on the mode of internalisation, we explored the native binding partners of haemoglobin in the mouse sera. Using affinity precipitation with haemoglobin Sepharose beads, we were able to pull down several binding partners of haemoglobin from the mouse sera, such as apolipoprotein A-1 (apoA1) (**Figure 6D**), a major protein component of high-density lipoprotein (HDL) particles in blood plasma. To confirm whether haemoglobin was integrated into the high molecular weight lipoprotein particles, we electrophoretically separated the plasma proteins under native conditions, and subsequently confirmed the presence of haemoglobin by immunodetection. Indeed, apart from a faint haemoglobin signal co-migrating with albumin, most of the haemoglobin signal was associated with the high molecular weight complexes of blood plasma (**Figure 6E**). The proteomics data further documented the internalisation of the HDL components *via* the Hp-independent uptake pathway (when parasites isolated from Hp-/- or Black mice were compared), but indicates a role of HpHbR in the uptake of HDL particles, as wild type parasites contained 36 HDL components, while HpHbR-KO parasites contained 16HDL components (**Supplementary Data S3**, **S5**).

## 3 Discussion

The presented findings reveal a complex and previously unexplored dimension to the interplay between haptoglobin (Hp), the blood plasma proteome, and the dynamics of *T. b. brucei* infection in a laboratory mouse model, disclosing a novel facet of the complex host-parasite interplay. Perhaps the most unexpected discovery is the capacity of trypanosomes to acquire and internalise haemoglobin in the absence of Hp, as it challenges the prevailing notion that Hp is a required binding partner for internalisation of haemoglobin by the parasites via their HpHbR (8). Previous work showed that the internalisation of fluorescently-labelled haemoglobin by the parasite was unequivocally conditioned by co-supplementation with Hp or an Hp-related protein (8). We argue that this experimental setup was based on a non-physiological assumption of the existence of free haemoglobin, which is non-existent in blood plasma, where extracellular haemoglobin readily associates with plasma components (15). Hence, our data are not in direct conflict with the earlier finding that Hp is essential for haemoglobin uptake (8), but rather extends the claim and contextualises it within the *in vivo* environment and dynamics. Since results from the *in vitro* binding experiments supported the notion that Hp promotes haemoglobin binding to HbHpR (7, 9, 16), which was further corroborated by the failure of HpHbR-KO bloodstream trypanosomes to acquire haem (8, 14), we interpret our data by assuming that haemoglobin is bound to other plasma proteins. Pull-downs with immobilised haemoglobin from the mouse blood plasma containing a negligible amount of Hp confirmed earlier observations that apolipoprotein-A1 is its predominant partner. Apolipoprotein-A1 is a dominant component of HDL nanoparticles, which contain more than 80 proteins in mice, including α and β haemoglobin chains (17).

The incorporation of haemoglobin into the HDL is likely to be apolipoprotein-A1-dependent, as their association can be prevented by apolipoprotein-A1 peptide mimetics (18). Indeed, haemoglobin binds apolipoprotein-A extremely efficiently (19). Apolipoprotein A1 can thus constitute a binding partner of haemoglobin with HDLs, which the parasites actively endocytose (20). In such a scenario, haemoglobin would be internalised, along with the HDL particles, by the parasite both through HpHbR-dependent and HpHbR-independent routes, as previously predicted (21). Combined, these observations reflect the complexity of haemoglobin fate when in blood, i.e. its complexing with Hp and clearance by CD163-mediated routes, as well as capture by the HDLs. Importantly, the HDL-associated haemoglobin may be internalised by *T. b. brucei* (22) and used for haem extraction to promote the parasite’s haem-dependent metabolic processes (14). This mode of internalisation of haemoglobin is likely complementary to its prevailing HpHbR-mediated acquisition. In humans, Hp is a natural antagonist of the HDL component, called trypanolytic factor 1 (TLF-1), which is toxic to *T. brucei* due to the presence of lytic apolipoprotein L1. The induction and upregulation of Hp during the acute phase reduces protection against trypanosomes. In contrast, Hp -/- mice showed higher TLF-1-mediated protection against *T. b. brucei* infection, suggesting that TLF-1 and Hp compete for the substrate in a dose-dependent manner (23).

The bioavailability of haem cofactors is critical for the regulation of trypanosomiasis in the vertebrate host. As shown recently, the absence of HpHbR in *T. b. brucei* resulted in a rapidly progressing parasitaemia caused by the absence of stumpy forms (14). Hence, due to the absence of Hp, we expected a similar parasitaemia surge to occur in Hp-/- mice. Surprisingly, the parasitaemia was comparable in both Hp-/- and Black mice with typical parasitic wave rises and falls. According to our measurements, regardless of the host’s genetic background, the parasites acquire haem *b.* As we have shown earlier (12) and as corroborated here, the haem chromatography profiles of the procyclic and bloodstream stages differ significantly, with haem *o* detected only in the latter, probably due to its immediate conversion to the final product. In contrast, haem *a* is not formed at all in the bloodstreams, while the haem *o* precursor accumulates (14). The *in vivo* system thus likely better reflects the complexity of the parasite - host interaction, including multiple routes of haem uptake. Accumulation of haem *o* in the bloodstream of *T. b. brucei* has not been reported so far and the role of this pigment remains enigmatic. Such plasticity and versatility in binding among the blood plasma molecules indicated in this work appears to be common and is not only specific for haemoglobin, upon Hp knock out, but also for haem, upon hemopexin (Hpx) knock out, whereby the haem buffering capacity of serum from adult Hp-/-Hpx-/- mice was found to be indistinguishable from that of control Hp+/+Hpx+/+ mice, suggesting that circulating proteins and/or macromolecules other than Hp and/or Hpx can scavenge labile haem (24).

The procyclic stage of *T. brucei* from the insect vector has a fully active mitochondrion and, therefore, contains more haemoproteins, such as subunits of the respiratory complexes (4), and takes up haem *via* the haem transporter HRG (25), which is well described in other haem auxotrophs such as ticks or *Leishmania* flagellates (26, 27). Since the bloodstream trypanosomes rely on glycolysis, with many mitochondrial haemoproteins downregulated or absent (25), the expression of HRG decreases in the mammalian host (25). Although in bacteria haem *o* can serve as a genuine cofactor of ubiquinol oxidases, eukaryotic alternative oxidases are di-iron proteins and mitochondrial terminal oxidases (COX) require haem *a.* In eukaryotes, haem *o* is thus known only as an intermediate of haem *a* biosynthesis (28). After uptake into the trypanosome cell, haem *b* is converted into haem *a* through the intermediate haem *o*, whose biosynthesis and role are poorly understood. However, the bloodstream *T. b. brucei* lacks a physiologically relevant level of COX (29) and, indeed, we did not detect haem *a*. Therefore, it is remarkable that a significant pool of acquired haem *b* is farnesylated. The mitochondrial biosynthesis of haem *o* and its likely relocation to the membranes indicates a signalling role. Why haem *o* does not accumulate in parasites in Hp -/- mice needs to be addressed in a future study.

The presented mouse model data cannot be fully extrapolated to humans because of inherent differences in Hp dynamics in their blood. Mice have, by default, very low levels of Hp in their bloodstream, in some strains even below the detection limit (30). In contrast, in human blood, Hp is a highly abundant plasma protein, present in about a gram per litre, which has profound consequences for the binding capacity of haemoglobin released into plasma during physiological and pathological haemolyses (10, 11). For these reasons, the role of mouse Hp in *T. b. brucei* infection might be conceptually questionable, but since elevated Hp levels are a concomitant feature of parasitaemia, we set out to explore its function in the Hp-/- mice. Human haemoglobin forms, with Hp, a high affinity HpHb complex (31), which is subsequently bound by a dedicated transmembrane receptor, CD163 (10). Recognition of the CD163-HpHb complex is necessary for its endocytosis and clearance from the bloodstream (10, 32). Similarly in mice, CD163 also recognises HpHb complexes but, unlike its human homologue, the mouse CD163 also binds extracellular haemoglobin, existing transiently to facilitate effective clearance of Hb (11). In fact, such removal of free haemoglobin from mouse blood is extremely efficient, with the capacity to clear 0.1 mg Hb per 10 g bodyweight (constituting 1 ‰ of the total Hb content in mice, i.e. a realistic estimate of the physiological situation) within minutes (11). Such an ability of mouse CD163 may have evolved due to the low default levels of its binding partner, Hp, to prevent toxic effects of extracellular haemoglobin by its swift clearance. Overall, the Hp-/- mouse, with a very short half-life of its free haemoglobin, provides a dynamic framework for the study of haemoglobin partitioning between *T. b. brucei* and its host.

The proteomic analysis determined broader changes in the plasma protein repertoire, extending beyond the expected absence of Hp in Hp-/- mice and known markers elicited by the *T. b. brucei* infection (33). The altered abundance of immunoglobulins, ferritin, and α-1 anti-trypsin, among others, suggests a compensatory response triggered by the ablation of Hp. It has been shown previously that the development of T- and B-cells is inhibited in mice subjected to this genetic manipulation, resulting in a significantly lower level of antigen-specific IgGs (34, 35). Thus, the haemoglobin-binding role of Hp is extended by its crucial contribution to the regulation of proliferation and functional differentiation of these immune system components. Indeed, our proteomics data of mouse sera also show substantially modulated levels of individual immunoglobulins, but the overall level of circulating IgGs appears unaffected. In contrast, Hp-/- mice show an increased concentration of ferritin heavy and light chains.

The highly significant inverse correlation between serum Hp and urinary (36) or epithelial ferritin (37) is likely a systemic response to the elevated levels of unbound haemoglobin, and thus of available haem. Additionally, we detected an increased level of gallectin-1, which is involved in pathogen recognition and the modulation of innate and adaptive host immune responses (38), including those against *Trypanosoma cruzi* (39). Moreover, it is worth noting that numerous host proteins, such as SAA1, SAA2, FABP, and AGP, are responsive (FC > 5) to *T. b. brucei* in both Hp-/- and Black mice. The SAA proteins are key components of the acute phase response following trauma, infection and other stimuli, during which, their serum levels rise dramatically (40). Due to their lipophilic nature, SAA proteins majorly partition into HDL particles (41).

In summary, our data challenge the conventional paradigm regarding the indispensability of Hp for successful *T. b. brucei* infection and, perhaps even more importantly, identify another element of the remarkable adaptability and flexibility of these blood parasites. We suggest that while the Hp-haemoglobin complexes appear transiently in the blood, they are internalised by *T. b. brucei* via HpHbR-mediated uptake. Displaying a longer half-life in blood, the lipoprotein complexes with Hb may thus represent a constant source of the haemoglobin-derived haem for trypanosomes.

## 4 Materials and methods

### 4.1 *T. b. brucei* cell growth and mouse infections

Bloodstream *T. b. brucei* 90-13 were cultivated at 37 °C in HMI-9 medium (Thermo Fisher), supplemented with 10% heat-inactivated fetal bovine serum. Cell densities were measured using the Z2 Coulter counter (Beckman Coulter). Black and Hp-/- mice (four to six weeks old) were injected intraperitoneally with 1 × 10^5^ 90-13 strain *T. b. brucei* cells. Infection was followed daily by dilution of tail snip blood in TrypFix buffer (3.7% formaldehyde, 1×SSC buffer) and manual counting of trypanosomes in a Neubauer hemocytometer.

### 4.2 Transgenic cell lines

HpHbR-KO cell lines were generated by successive deletion of alleles with pPuro-KO or pHygro-KO, and pPhleo-KO constructs. Trypanosomes were transfected with linearized plasmid DNA (10 μg). Briefly, a total of 1 × 10^7^ *T.b. brucei* cells was harvested by centrifugation at 1000 × *g* at 4°C for 10 min and washed 1 time with PBS. Cells were resuspended in 100 μL of AMAXA Human T-cell solution and electroporated with AMAXA Nucleofactor apparatus. Predefined program X-001 was used for electroporation. Selection markers were applied 6 h post-transfection, and clones were generated by limited dilution. The Hp-/- mouse strain was previously generated by allelic disruption through homologous recombination (Lim et al., 1998), and the heterozygotes were crossed by us to generate the full KO mouse strain.

### 4.3 Parasite isolation for downstream analyses (proteomics and EM)

Before the trypanosomes were recovered from the bloodstream, the mice were euthanised with Narketan/Xlazine solution and heparin (500 U per mouse) was administered intraperitoneally to prevent blood clotting. The parasites were separated from the erythrocytes on a diethylaminoethyl (DEAE) cellulose column according to a standard protocol. The purified trypanosomes were washed once with PBS and frozen at -80°C for MS/MS and HPLC analysis.

Before and during infection, blood was collected from the tail tip of the mice (9 uL) and mixed with 1uL of 109 mM sodium citrate (Sigma Aldrich, C8532), centrifuged at 1000 × *g* for 5 minutes and the upper layer (plasma) was frozen at -80°C for MS/MS and Western blot analysis.

### 4.4 PCR and western blot analysis

Total DNA was extracted from the blood of Black and Hp-/- mice using a GeneAll kit (Exgene Clinic SV) and used as a template for PCR analysis of mouse haptoglobin (NM_017370) and β−actin (NM_007393) as a control with the following primers: b-actin_musFW1: GGCCGGGACCTGACAGACTACCTC, b-actin_musRV1:GTCACGCACGATTTCCCTCTCAGC, b-actin_musRV2: ACATCTGCTGGAAGGTGGACAGTG, haptoglobin_musFW1: TGTTGTCACTCTCCTGCTC, haptoglobin_musRV1: TCACCCATTGCTTCTCGTC, haptoglobin_musRV2: CACACACTGCCTCACATTC.

Lysates from 5 × 10^6^ *T. b. brucei* cells were separated on a 12% SDS-polyacrylamide gel, transferred to a PVDF membrane, and probed with the polyclonal anti-Hp (ab256454, Abcam) and anti-Hb (MA5-32328, Invitrogen) antibodies at 1:2000 dilutions and with the monoclonal anti-alpha-tubulin (T9026, Sigma Aldrich) at 1:5000 dilution as a loading control. The anti-mouse and anti-rabbit secondary antibodies were horseradish peroxidase conjugated and detected by ECL substrate (#1705061, BioRad). The images were detected with BioRad Image Lab software.

### 4.5 Hemoglobin pull-down and pore-limit native page electrophoresis

CNBr-activated Sepharose™ 4B beads were incubated with haemoglobin in PBS and subsequently blocked with 1M Tris solution. These beads were incubated with sera C3N mice and subsequently washed with PBS before Urea (1M and 8M) elutions. Blank elution was performed on haemoglobin beads incubated with PBS only, without mouse sera.

Sera from mice were separated in pore-limit native PAGE in Tris-Borate-EDTA (TBE) buffer (0.09M Tris, 0.08M boric acid, 2mM EDTA). Samples were loaded in the running buffer supplemented with 10% (vol/vol) glycerol and 0.001% (w/vol) bromophenol blue. Samples were run in 4−16% Bis-Tris gel (Invitrogen) at 150 V in a cold room for 12 hr. Proteins were transferred onto nitrocellulose using a Trans-Blot Turbo system (BioRad) and used for Western blot analyses as described above and stained with Amido Black to visualise proteins.

### 4.6 Haem quantification using high-performance liquid chromatography

Bloodstream cells were harvested by centrifugation at 1000 × *g* at 4°C for 10 min and washed 3 times with PBS on ice. Cells were resuspended in 30 μL H_2_O, extracted with 200 μL acetone/0.2% HCl, and the supernatant was collected after centrifugation at 1000 × *g* at 4°C for 5 min. The pellet was resuspended in 100 μL acetone/0.2% HCl and centrifuged as described above. Both supernatants were combined, and 150 μL of each sample was immediately injected into a high-performance liquid chromatography system (Infinity 1200, Agilent Technologies) and separated using a reverse-phase column (4 μm particle size, 3.9 × 75 mm) (Waters) with 0.1% trifluoroacetic acid and acetonitrile/0.1% trifluoroacetic acid as solvents A and B, respectively. Pigments were eluted with a linear gradient of solvent B (30–100% in 12 min) followed by 100% of B at a flow rate of 0.8 mL/min at 40°C. Haem species were detected by diode array detector (Infinity 1200, Agilent Technologies) and identified by retention time and absorbance spectra according to control extracts (see **Figures 5B, C**).

### 4.7 Mass-spectrometry proteomic analysis

Blood from mouse tails (9 μL) was collected, mixed with 1 μL heparin and centrifuged at 4000x g for 5 minutes. The supernatant containing the blood plasma was collected and frozen at -80°C until analysis. *Trypanosoma* cell pellets were sonicated and treated with Benzonase Nuclease (Merck 70664-3) to isolate proteins. Cell proteins or mouse sera corresponding to approximately 100 microgram of protein were processed according to the SP4 glass bead-free protocol (42). Briefly, samples were solubilised with SDS [final concentration 1% (w/v)] in 100 mM TEAB (triethylammonium bicarbonate), reduced with TCEP [tris(2-carboxyethyl)phosphine], alkylated with CAA (chloroacetamide), and digested with trypsin (Trypsin Gold, Mass Spectrometry Grade, Promega V5280) over-night at 37°C at 1:50 ratio (trypsin:protein). Samples were desalted on Empore C18 columns, dried in a Speedvac and dissolved in 0.1% TFA + 2% acetonitrile. About 500 ng of peptide digests were separated on a 50 cm C18 column (EASY-Spray ES903, Thermo Fisher Scientific) using 120 min (sera 90 min) elution gradient (Dionex Ultimate 3000, flowrate 300 nL/min) and analysed in a DIA mode on an Orbitrap Exploris 480 mass spectrometer equipped with a FAIMS unit (Thermo Fisher Scientific). The resulting raw files were processed and visualised in Spectronaut 18.6 (Biognosys) software using the default setting with Precurson and Protein Q-value and PEP cutoff set at 0.01. Proteome fasta sequences used were: UP000000589_10090.fasta (mouse, UniProt release 2023_01) and TriTrypDB-64_TbruceiTREU927_AnnotatedProteins.fasta (tritrypdb.org, release 64).

### 4.8 Transmission electron microscopy and immunogold labelling

Cells were fixed in 4% formaldehyde with 0.1% glutaraldehyde in 0.1 M HEPES pH 7.2 for 1 h at room temperature (RT). After washing in HEPES buffer, samples were embedded in 15% gelatin, cryoprotected in 2.3 M sucrose for 72 h at 4°C, and frozen by plunging into liquid nitrogen. Ultrathin cryosections were cut at -100°C, and pick-up with 1.15 M sucrose/1% methylcellulose solution (25 cp, Sigma). Sections were incubated for 1 h at RT in 1% fish skin gelatin (FSG) with 0.05% Tween20 and labelled with monoclonal rabbit IgG directed to haemoglobin (SN70-09, Invitrogen) diluted 1:30 in FSG for 1 h at RT. After washing in FSG, the sections were incubated with protein A coupled with 10 nm gold nanoparticles (British BioCell International) diluted 1:50 in FSG for 30 min at RT. Sections were washed in HEPES, postfixed for 5 min in 1% glutaraldehyde diluted in 0.1 M HEPES, washed in dH_2_O, and then contrasted/embedded in a mixture of 2% methylcellulose and 3% aq. uranyl acetate solution (9:1). Samples were observed using a JEM 1400 TEM (JEOL) equipped with a CMOS camera XAROSA (EMSIS GmbH). Control sections were incubated without the primary antibody.

### 4.9 Statistical analysis

Statistical significance of differences were analyzed for two datasets (knock-out and wild-type, or infected and non-infected) using GraphPad Prism 10 (GraphPad Software, CA) employing the t-test with *p* values < 0.05 considered as significant, with detailed descriptions in Figure legends.

## Data availability statement

The datasets presented in this study can be found in online repositories. The names of the repository/repositories and accession number(s) can be found below: PXD052778 (PRIDE).

## Ethics statement

The research on mice was approved by the Czech Ministry of Agriculture (project No. 28/2016 and 56-2022-P). All experimental procedures complied with the Czech law about Protection of Animals Against Cruelty (Act No. 246/1992, Decree No. 419/2012). The study was conducted in accordance with the local legislation and institutional requirements.

## Author contributions

EH: Conceptualization, Data curation, Formal analysis, Investigation, Methodology, Writing – review & editing. MVr: Data curation, Formal analysis, Investigation, Methodology, Writing – review & editing. MT: Investigation, Methodology, Writing – review & editing. ES: Investigation, Methodology, Writing – review & editing. JPi: Investigation, Methodology, Writing – review & editing. ZV: Investigation, Methodology, Writing – review & editing. MVa: Formal analysis, Investigation, Methodology, Resources, Visualization, Writing – review & editing. RS: Data curation, Formal analysis, Investigation, Methodology, Visualization, Writing – review & editing. JL: Formal analysis, Funding acquisition, Project administration, Resources, Supervision, Writing – review & editing. JPe: Conceptualization, Formal analysis, Funding acquisition, Investigation, Project administration, Writing – original draft, Writing – review & editing.
