# Supplementary material for: Haptoglobin is dispensable for haemoglobin uptake by Trypanosoma brucei
Source: Front Immunol. 2024 Jul 18;15:1441131. doi: 10.3389/fimmu.2024.1441131 (PMC11304504; doi:10.3389/fimmu.2024.1441131)
Supplement: Supplementary file 2 [file DataSheet_2.docx]

**Supplementary data**

**Haptoglobin is dispensable for haemoglobin uptake by *Trypanosoma brucei***

Eva Horáková^1,2^, Marek Vrbacký^3^, Martina Tesařová^1^, Eva Stříbrná^1^, Jan Pilný^2^, Zuzana Vavrušková^1^, Marie Vancová^1,4^, Roman Sobotka^2,4^, Julius Lukeš^1,4,*^, & Jan Perner^1,*^

^1^ *Institute of Parasitology, Biology Centre, Czech Academy of Sciences, České Budějovice, Czech Republic*

^2^ *Centre Algatech*, *Institute of Microbiology, Czech Academy of Sciences, Třeboň, Czech Republic*

^3^ *Institute of Physiology, Czech Academy of Sciences, Prague, Czech Republic*

^4^ *Faculty of Science, University of South Bohemia, České Budějovice, Czech Republic*

* correspondence: jula@paru.cas.cz, [perner@paru.cas.cz](mailto:perner@paru.cas.cz)


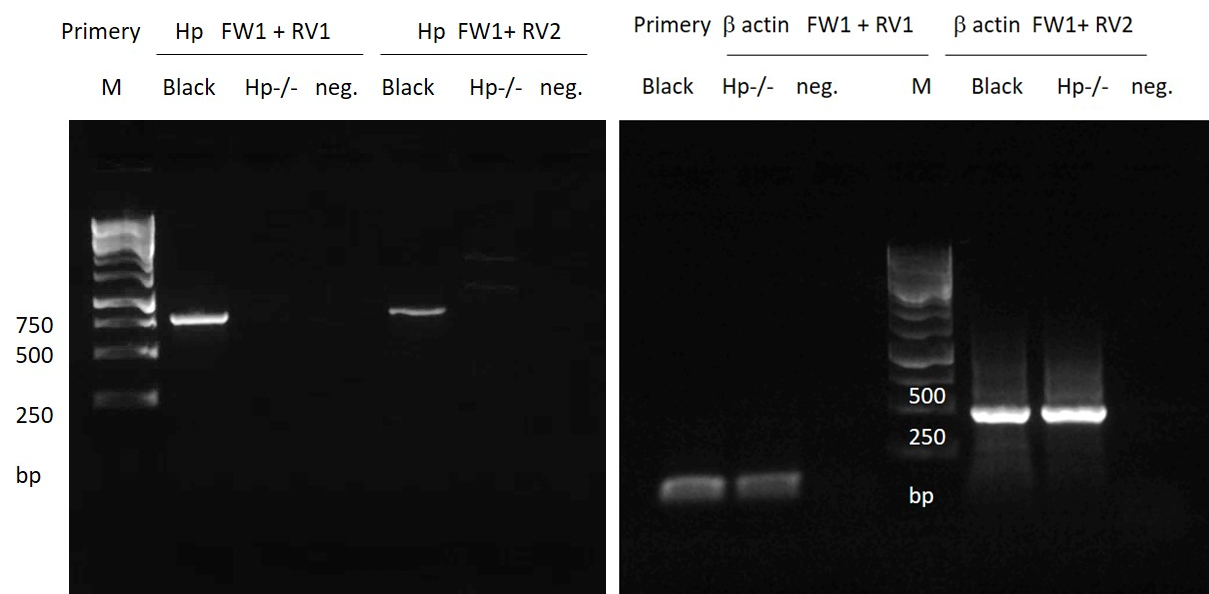


**Supplementary Figure S1. Validation of *haptoglobin* gene knock out.** PCR and ethidium-bromide stained agarose gel electrophoresis was conducted on DNA isolated from mice blood (Hp-/- and Black strains), using two sets of *haptoglobin*-targeted primers (FW1+RV1; FW1+RV2). Similarly, two primer sets were used for *beta actin* control gene (FW1+RV1; FW1+RV2). Neg. indicates No Template Control.

**
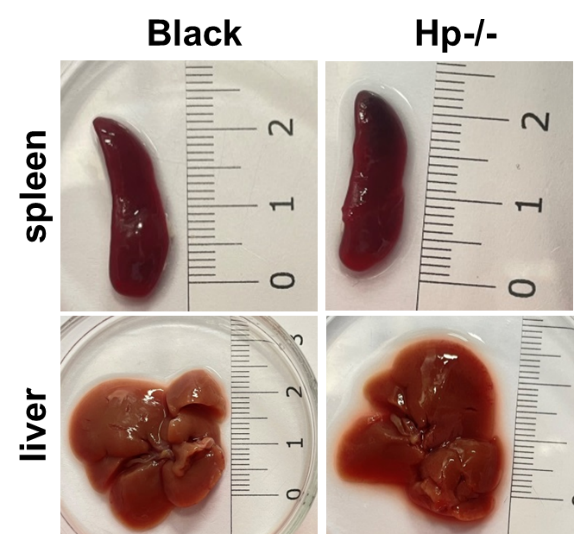
**

**Supplementary Figure S2. A representative image of spleen and liver from Hp-/- and Black mice upon *T. b. brucei* infection.** Photographs of spleen and liver from Black and haptoglobin deficient (Hp-/-) mice upon wild type *T. b. brucei* infection (4 days post infection). A supporting image for Figure 3B, C.

**
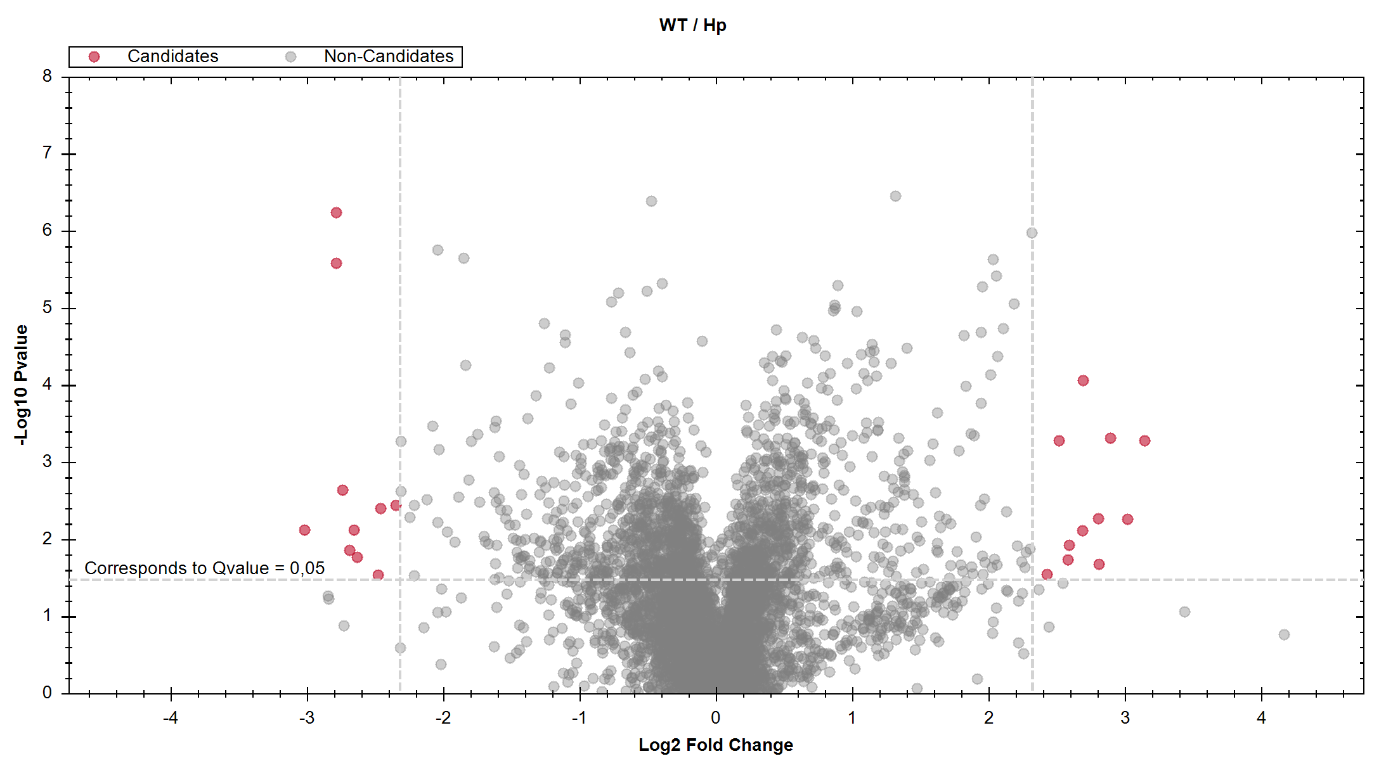
Supplementary Figure S3. Volcano plot of wild type *T. b. brucei* proteome showing statistical differences between parasites isolated from Hp-/- and control mice.** A volcano plot of proteins identified in the purified pellets of parasites (parasite and mice proteins) isolated from the Hp-/- and Black mice 12 days post-infection, with statistical significance of Qvalue = 0.05 and Log2 Fold Change 2.32 (equals to absolute Fold Change > 4.99).

**
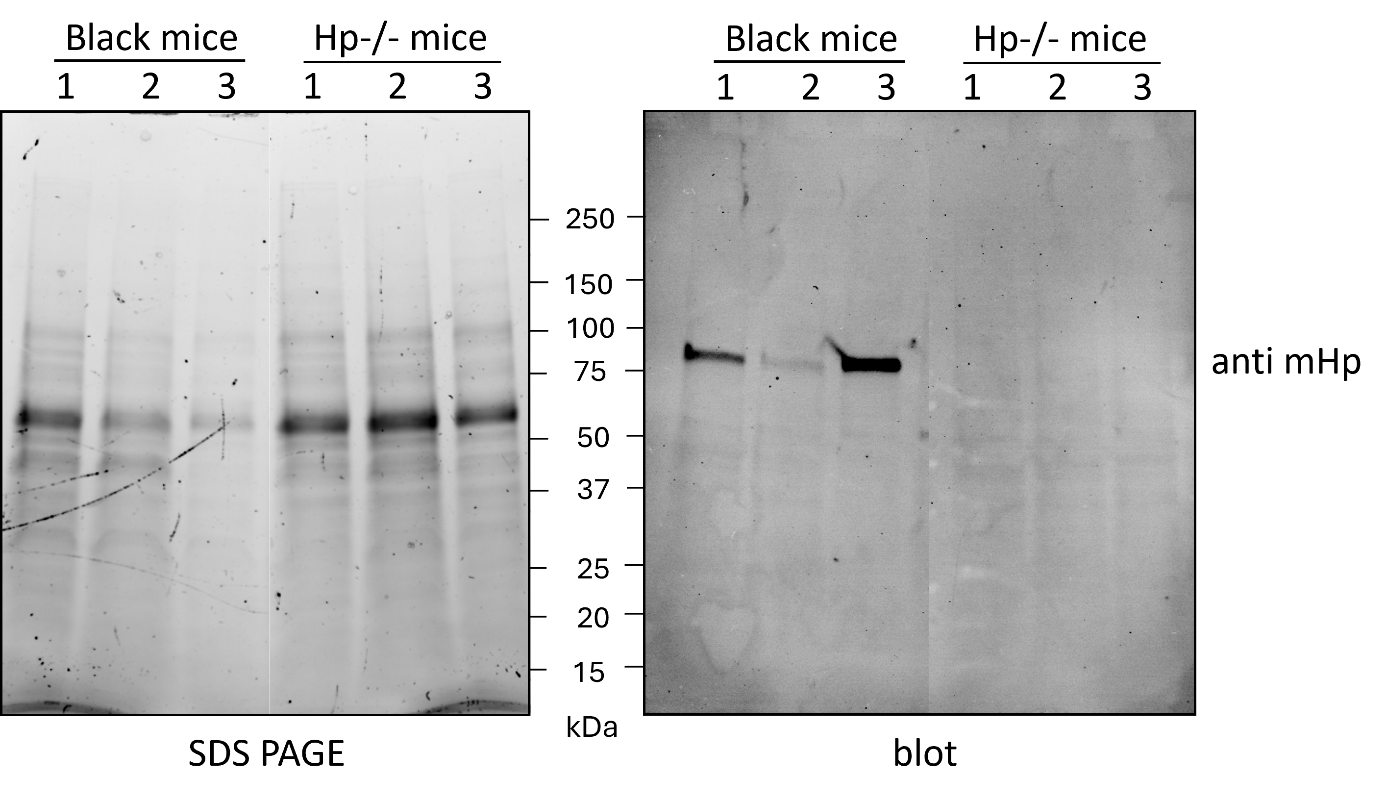
**

**
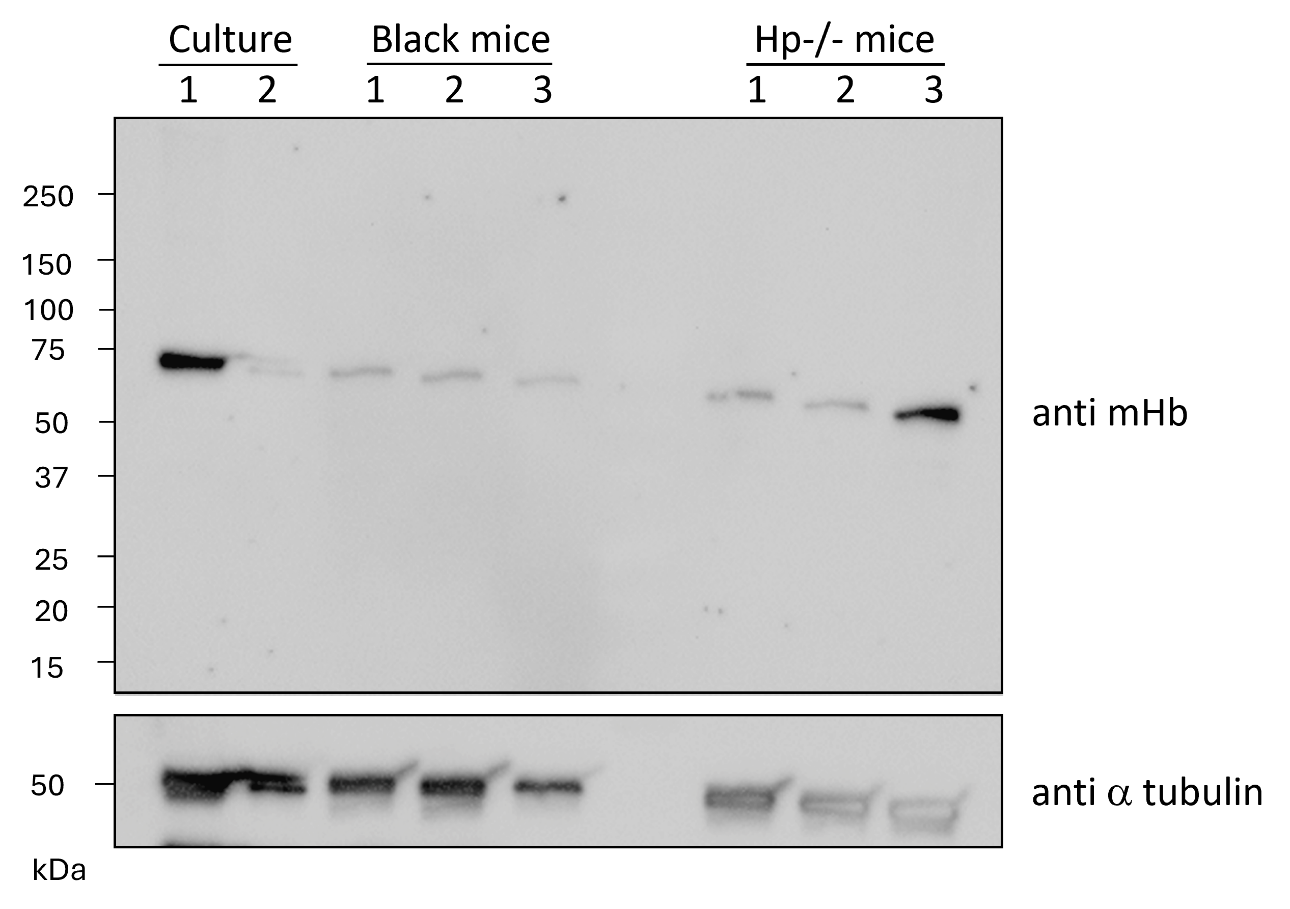
Supplementary Figure S4. Western blot confirms similar haemoglobin levels in parasites isolated from Black mice or haptoglobin deficient (Hp-/-) mice.** *T. b. brucei* homogenate was separated on SDS-PAGE and, upon blotting, incubated with commercial antibodies recognising mouse haptoglobin (mHp; upper panel) or haemoglobin (mHb; lower panel) respectively. Parasites were harvested from Black mice or haptoglobin deficient (Hp-/-) mice on the fifth day post-infection. Cultured bloodstream parasites (90-13) and alpha tubulin detection were used as controls.


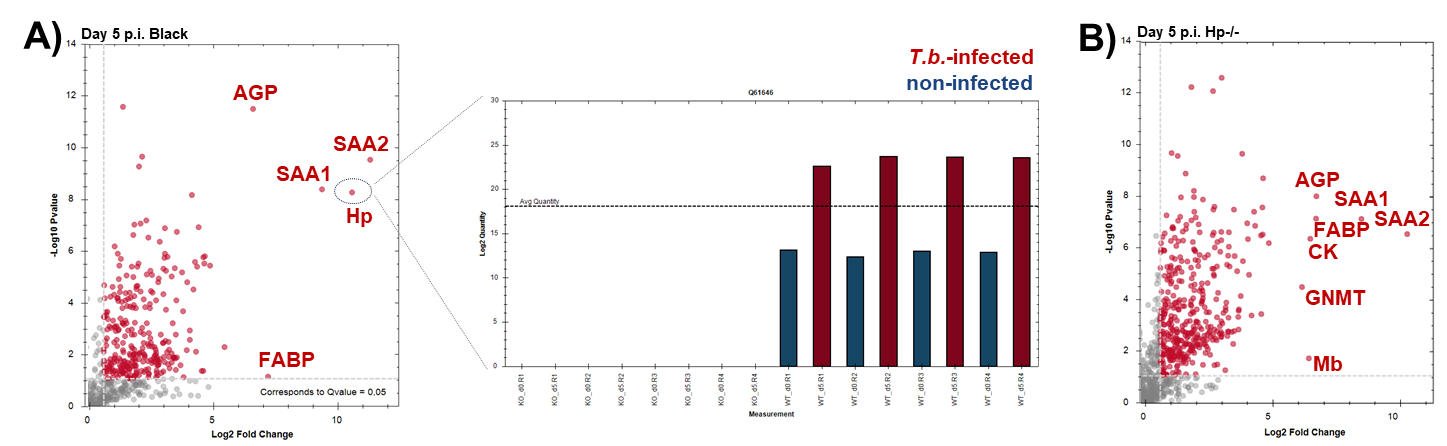


**Supplementary Figure S5. A, B)** Volcano plots of induced changes in mouse (A, Black strain; B, Hp-/- strain) plasma proteome in response to HpHbR-KO *T. b. brucei* parasites with statistical significance of Qvalue = 0.05 and Log2 Fold Change > 0.58. Note, in the inset of panel A, that haptoglobin (Hp) is induced by HpHbR-KO *T. b. brucei* infection (red bars) compared to uninfected Black mice (blue bars); no Hp peptides are retrieved from Hp-/- mice (first eight samples of panel A inset) with no protein appearance in panel B.

**
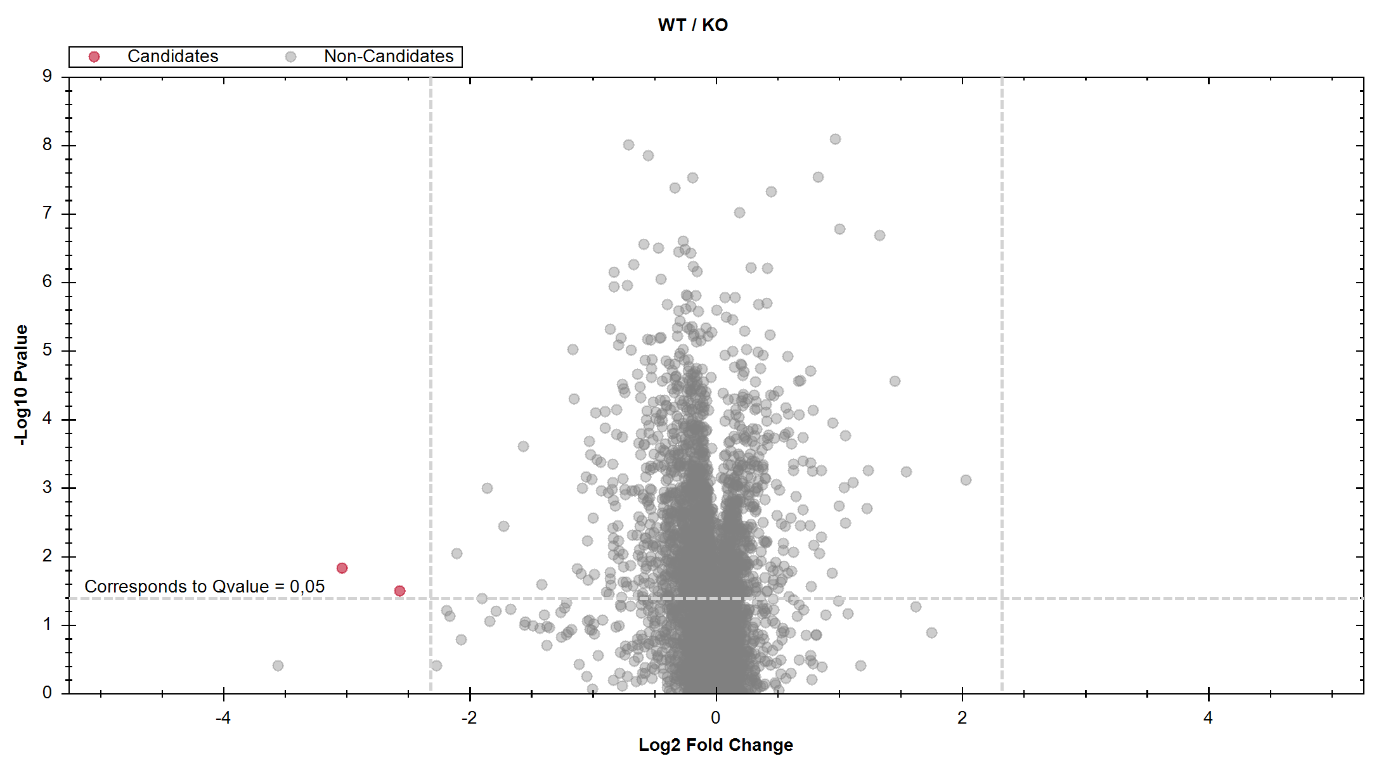
**

**Supplementary Figure S6. Volcano plot of HpHbR-KO *T. b. brucei* proteome showing statistical differences between parasites isolated from Hp-/- and control mice.** A volcano plot of proteins identified in the purified pellets of parasites (parasite and mice proteins) isolated from the Hp-/- and Black mice 5 days post-infection, with statistical significance of Qvalue = 0.05 and Log2 Fold Change 2.32 (equals to absolute Fold Change > 4.99). The red arrow points towards mouse haemoglobin alpha subunit.
